# Improving sequence-based modeling of protein families using secondary-structure quality assessment

**DOI:** 10.1093/bioinformatics/btab442

**Published:** 2021-06-12

**Authors:** Cyril Malbranke, David Bikard, Simona Cocco, Rémi Monasson

**Affiliations:** Laboratory of Physics of the Ecole Normale Superieure, PSL Research, CNRS UMR 8023, Sorbonne Université, Université de Paris, Paris, France; Synthetic Biology, Microbiology Department, Institut Pasteur, Paris, France; Synthetic Biology, Microbiology Department, Institut Pasteur, Paris, France; Laboratory of Physics of the Ecole Normale Superieure, PSL Research, CNRS UMR 8023, Sorbonne Université, Université de Paris, Paris, France; Laboratory of Physics of the Ecole Normale Superieure, PSL Research, CNRS UMR 8023, Sorbonne Université, Université de Paris, Paris, France

## Abstract

**Motivation:**

Modeling of protein family sequence distribution from homologous sequence data recently received considerable attention, in particular for structure and function predictions, as well as for protein design. In particular, direct coupling analysis, a method to infer effective pairwise interactions between residues, was shown to capture important structural constraints and to successfully generate functional protein sequences. Building on this and other graphical models, we introduce a new framework to assess the quality of the secondary structures of the generated sequences with respect to reference structures for the family.

**Results:**

We introduce two scoring functions characterizing the likeliness of the secondary structure of a protein sequence to match a reference structure, called Dot Product and Pattern Matching. We test these scores on published experimental protein mutagenesis and design dataset, and show improvement in the detection of nonfunctional sequences. We also show that use of these scores help rejecting nonfunctional sequences generated by graphical models (Restricted Boltzmann Machines) learned from homologous sequence alignments.

**Availability and implementation:**

Data and code available at https://github.com/CyrilMa/ssqa

**Supplementary information:**

Supplementary data are available at *Bioinformatics* online.

## 1 Introduction

Considerable efforts were devoted over the past decade to the modeling of protein families from homologous sequence data, taking advantage of the tens of millions of available sequences in databases such as UniProt (The UniProt Consortium, 2019) or PFAM (Bateman *et al.*, 2002). Among sequence-based models, graphical models, in particular direct coupling analysis (DCA), emerged as simple and effective Bayesian inference approaches capturing essential statistical properties of residues in sequence data, such as their conservation and pairwise correlations, see Cocco *et al.* (2018) for a review. DCA outputs a set of statistical pairwise couplings, which are informative about the contact map of the single or multiple folds (Malinverni *et al.*, 2015; Weigt *et al.*, 2009) characterizing the family, or about the protein interactions with its partners (Bitbol *et al.*, 2016). In addition, DCA defines a likelihood over the sequence space, which can be used to predict the effects of mutations to a natural sequence in comparison to mutagenesis experiments (Figliuzzi *et al.*, 2016; Hopf *et al.*, 2017), or can be sampled to design *de novo* synthetic proteins, whose viability can be assessed *in vivo* (Russ *et al.*, 2020).

Despite these successes it remains unclear what aspects of the structural, functional and evolutionary constraints acting on protein sequences are adequately captured by such sequence-based models, and, conversely, what features are inappropriately accounted for. Here, we introduce a method to assess the compatibility of these models with the secondary structure elements common to the family. The goal of our secondary-structure quality assessment (SSQA) method is twofold. First, we may use SSQA to *a posteriori* test the validity of sequence-based model predictions, as failure to preserve the secondary structure of a protein is likely to result in a loss of its functionalities. Second, SSQA can be used to guide protein design by helping the production of sequences with adequate secondary structures.

**Fig. 1.**
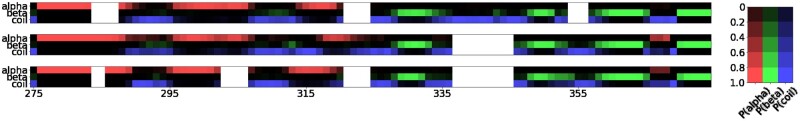
Profiles of predicted secondary structures (*α*-helix, *β*-strand or coil, see probability values on the right color scale) computed with NetSurfP2 (Klausen *et al.*, 2018) for three sequences of the betalactamase family (PF00144, aligned sequences: YFEW_ECOLI/43-414, P74474_SYNY3/27-387, P94288_BACCE/53-388). Alignment (induced from the PFAM alignment) between the three sequences had a length of 398. For the sake of clarity, only positions 275–375 of the alignments are shown. White positions correspond to gaps in the alignment. Note the similarities between the three secondary structures

SSQA aims at estimating the similarity between the putative secondary structure associated to a given sequence and a reference structure associated to the protein family. This task is analogous to (tertiary)-structure quality assessment, which has received sustained attention in the past years (Baldassarre *et al.*, 2021; Derevyanko *et al.*, 2018). Our focus on secondary structure is motivated by several reasons. Secondary structure is known to be largely conserved in protein families (Fig. 1), and is therefore a reliable signature of family membership. In addition, state-of-the-art algorithms for secondary structure predictions, such as JPred4 from Drozdetskiy *et al.* (2015), NetSurf0-2.0 from Klausen *et al.* (2018), Wang *et al.* (2016) or Asgari *et al.* (2019) reach very high accuracy levels (85–90%). The availability of computationally fast and reliable tools is necessary to the implementation of our quality assessment approach.

Our paper is organized as follows. We briefly review graphical models, in particular Restricted Boltzmann Machines, an unsupervised learning framework that encompasses DCA by including high-order couplings between residues in Section 2.1, as well as secondary structure inference algorithms in Section 2.2. SSQA with its different formulations are presented in Section 3. Results on the ability of SSQA to improve functionality/activity prediction are reported in Sections 4.1 and 4.2. We then show how protein data-driven design (Section 4.3) based on Restricted Boltzmann Machines can be enhanced with SSQA. Conclusive remarks can be found in Section 5.

## 2 Background

### 2.1 Graphical models for sequence distributions and Restricted Boltzmann Machines

We will consider hereafter protein sequence distributions P(x) expressed by graphical models, where $x=\{x_{i}\}$ denotes the sequence of amino acids. A well-known example of graphical model is the so-called DCA, for which

(1) $$P(x)=\frac{1}{Z}e^{-E_{\mathrm{DCA}}(x)}\;\text{with}\;Z=\sum_{x\prime }e^{-E_{\mathrm{DCA}}(x\prime )}\;,$$

and the energy function is

(2) $$E_{\mathrm{DCA}}(x)=-\sum_{i}g_{i}(x_{i})-\sum_{i<j}J_{ij}(x_{i},x_{j})\;.$$

The set of parameters $g_{i}(x)$ and $J_{ij}(x,y)$ are inferred so that the 1- and 2-point statistics, revealing conservation and coevolution in homologous sequence data match the ones of the model distribution. DCA was shown to be successful for extracting structural information about the 3D conformation of the protein and for designing new functional proteins through the sampling of P(x) (see Russ *et al.*, 2005, 2020).

In this work, we will consider another class of graphical models called Restricted Boltzman Machine (RBM, see Salakhutdinov (2008) for an overview), which encompass DCA and may also express interactions of order ≥3 between residues in the sequence. RBM was recently shown to be powerful to model amino-acid sequence distributions (Bravi *et al.*, 2020; Tubiana *et al.*, 2019). Briefly speaking, RBM is joint probabilistic models on bipartite graphs, with one layer carrying the sequences *x* and another layer, the representations $h=\{h_{\mu }\}$. The energy function for *x*, *h* is

(3) $$E_{\mathrm{RBM}}(x,h;W)=-\sum_{i}g(x_{i})+\sum_{\mu }U(h_{\mu })-\sum_{i,\mu }W_{i\mu }(x_{i})h_{\mu }\;.$$

This energy defines the joint distribution of sequences and representations

(4) $$P(x,h)=\frac{1}{Z}e^{-E_{\mathrm{RBM}}(x,h)}\;.$$

The interactions *W* and the potential *g* acting on the input units are similar to position weight matrices, and are learned through maximization of the marginal distribution P(x) of the sequences *x* in the training dataset. To do this, methods such as Persistent Contrastive Divergence (PCD) can be used (see Tieleman, 2008; Tubiana *et al.*, 2019). The potentials acting on the hidden model *U* are chosen to be quadratic: $U(h_{\mu })=\frac{1}{2}h_{\mu }^{2}$. Note that it is possible to learn the potentials *U* (see Tubiana *et al.*, 2019), an option that was not retained here.

The joint probability in (4) also allows one to define the conditional probabilities P(x|h) and P(h|x). Due to the bipartite nature of the interaction graph, these conditional probabilities are factorized, which makes sampling fast and easy. With our choice of a quadratic potential over the representation units, we get the following conditional probabilities for, respectively, representational and sequence units:

(5) $$P(h_{\mu }|x)=\frac{1}{\sqrt{2\pi }}\text{exp}\;[-\frac{1}{2}{\left(h_{\mu }-\sum_{i}W_{i\mu }(x_{i})\right)}^{2}],$$

 

(6) $$P(x_{i}|h)=\text{softmax}[g_{i}(x_{i})+\sum_{\mu }W_{i\mu }(x_{i})h_{\mu }].$$

Alternating sampling of the representation and the sequence layers provide an efficient Gibbs procedure to sample P(x), see Algorithm 1.

**Algorithm 1 Gibbs sampling through RBM**

1: **function**  Sampling RBM(*X*, Ξ)

2:   Pick $x^{\left(0\right)}$ in the set of natural sequences (NAT)

3:   **for**  ξ∈[1,Ξ]  **do**

4:    Sample $h^{\left(\xi \right)}$ following $P(h^{\left(\xi \right)}|x^{\left(\xi -1\right)})$

5:    Sample $x^{\left(\xi \right)}$ following $P(x^{\left(\xi \right)}|h^{\left(\xi \right)})$

### 2.2 Secondary structure: definition and inference

Secondary structure is the three-dimensional form taken by a protein on local scales. The two main secondary structural motifs are *α*-helices (with H-bonds between amino acids that are 3–4 residue apart along the sequence) and *β*-sheets (multiple strands connected by at least 3 H-bonds). We thus represent the secondary structure of a protein by a sequence of a 3-class classification following the primary structure (chain of residues): *α*-helix, *β*-strands, or ‘coil’ if the residue is part of a disordered segment or an irregular structure. We will also consider more detailed classifications involving eight classes, see Kabsch and Sander (1983). This classification includes three subclasses for α-helix (3-turn helix, 4-turn helix and 5-turn helix), two subclasses for β-strand (isolated *β*-bridge or extended strands) and three subclasses for coil (turn, bend or other).

Hereafter, we consider models, denoted by M, allowing us to estimate the probability for each site *i* in a sequence *x* to be part of a secondary-structure class, e.g. *α*-helix, *β*-strand or *coil*. These models can be very simple (based on statistics of amino acids), but most successful algorithms now rely on Deep Learning, including one-dimensional convolutional or recurrent neural networks, in particular LSTM (Hochreiter and Schmidhuber, 1997). Many of these models are proposed in the literature (Asgari *et al.*, 2019; Klausen *et al.*, 2018). The most competitive algorithms enrich the sequence of amino acids *x* with hidden Markov model (HMM-er) profiles computed from homologous sequences.

In the present work, we focused on $M_{1}$, an adapted network based on NetSurfP2-0 from Klausen *et al.* (2018). Our implementation trained on 10.384 sequences with MMseqs profiles reaches 85% accuracy on validation set of 500 sequences, 84.1% accuracy on TS115 dataset (Yang *et al.*, 2018) and 83.5% accuracy on CB513 dataset (Cuff and Barton, 1999) with a relatively light architecture. For training and validation, we used training and testing datasets from Klausen *et al.* (2018). Both models relied on HMM profiles built through HHsuite (Steinegger *et al.*, 2019).

In Figure 1, we show the probability maps computed with $M_{1}$ for three sequences of the betalactamase family (PFAM family PF00144). Observation of these profiles on various sequences and families suggests that aligned residues are likely to be part of the same secondary-structure class. In addition, sequences from the same family are likely to have very similar structures, following one or several patterns. As we show in Supplementary Section S2, errors in predictions are often encountered at the boundary between two distinct classes in the secondary structure (*border errors*) or when errors are made in the prediction, true labels often have a likelihood that is not negligible (*weak errors*). These two kind or errors made by predictors are common and we may then want to build a score that is robust to it.

## 3 Material and methods

In this section, we propose two ways of assessing the quality of the secondary structure (with respect to a reference secondary structure). Both rely on building a bag of local features from a protein sequence focused on secondary structure. Dot Product (DP) defines a bag of many raw features (one for each residue of a sequence), quickly computed and fully relying on alignments of the sequences. Pattern Matching (PM) produces a bag of few refined features (number of secondary structure motifs in the sequence), that require more computation time (quadratic in sequence length) and does not necessarily rely on alignments. Given the refinement of the PM features we expect that use of the corresponding features will require less (expensive) annotation data than DP.

### 3.1 Conditional distribution of secondary structures

Let *x* be a protein sequence of length *n*. Its secondary structure *s* is a string of length *n* taking value in $H_{n}={\left\{\alpha -\text{helix},\beta -\text{strand},\text{coil}\right\}}^{n}$, with $s_{i}=\alpha -\text{helix},\beta -\text{strand},\text{coil}$ if the residue *i* is part of, respectively, an *α*-helix, a *β*-strand, a disorganized segment (‘coil’). In the next part, we will also use the DSSP (Kabsch and Sander, 1983) classification with eight classes of secondary structure motifs.

Let us consider a model M for secondary structure inference from an amino-acid sequence. Given a sequence *x*, M returns a probability vector for each residue, that we can define as $P^{x}\in {\left[0,1\right]}^{n\times 3}$ where $P_{i,\hat{s}}^{x}=P(s_{i}=\hat{s}|x,M)$ with $\sum_{\hat{s}}P(s_{i}=\hat{s}|x,M)=1$. We may then introduce ℓ for any secondary structure *s* in $H_{\mathrm{n,}}$
 

(7) $$\ell^{x}(s)=\prod_{i}P_{i,s_{i}}^{x}=\prod_{i}P(s_{i}|x,M).$$

In an approximation in which secondary-structure symbols are independent, $\ell^{x}(s)$ represents the probability that *x* has secondary structure *s*, P(s|x,M). Distribution in (7) neglects the presence of correlations between sites, and will be used for the sake of mathematical tractability.

### 3.2 DP features

Let us consider for each *i* the probability vector of the secondary structure at residue *i*, $P_{i}^{x}=P(s_{i}=•|x)$. We compare the distribution of probabilities of two sequences *x* and *x*_0_ at residue *i* through

(8) $${\text{DP}}_{i}(x,x_{0})=\frac{\left(P_{i}^{x}|P_{i}^{x_{0}}\right)}{||P_{i}^{x}|{|}_{2}\cdot ||P_{i}^{x_{0}}|{|}_{2}}\;,$$

where (·|·) denotes the DP between two vectors, and $||\cdot |{|}_{2}$ the *L*_2_ norm: $||x|{|}_{2}={\left(\sum_{j}x_{j}^{2}\right)}^{\frac{1}{2}}$. ${\text{DP}}_{i}$ is a similarity measure between the two secondary structures associated to the sequences *x* and *x*_0_, equivalent to the cosine of the angle between their two associated vectors. The higher it is the more likely their secondary structures will coincide on site *i*, with ${\text{DP}}_{i}(x,x_{0})=1\Leftrightarrow P_{i}^{x}=P_{i}^{x_{0}}$. Low values of DP result, on the contrary, from discrepancies between the local predicted secondary structures, e.g. ${\text{DP}}_{i}(x_{1},x_{0})\approx 0.105$ for $P_{i}^{x_{1}}=(0.1,0.8,0.1)$ and $P_{i}^{x_{0}}=(0.1,0.1,0.8)$.

It is also possible to extend the definition of DP above to compare one sequence, say, *x*_1_, to a set of sequences, say, $X_{0}$ (including *N* sequences):

(9) $${\text{DP}}_{i}(x,X_{0})=\frac{\frac{1}{N}\sum_{x_{0}\in X_{0}}\left(P_{i}^{x}|P_{i}^{x_{0}}\right)}{||P_{i}^{x}|{|}_{2}\cdot ||\frac{1}{N}\sum_{x_{0}\in X_{0}}P_{i}^{x_{0}}|{|}_{2}}$$

Since the reference sequence, *x*_0_, or set of sequences, $X_{0}$, are fixed in practice, we now on simplify the notation ${\text{DP}}_{i}(x,X_{0})$ or ${\text{DP}}_{i}(x,x_{0})$ to ${\text{DP}}_{i}(x)$. These features will be later referred to as the DP features.

### 3.3 PM features

**Fig. 2.**
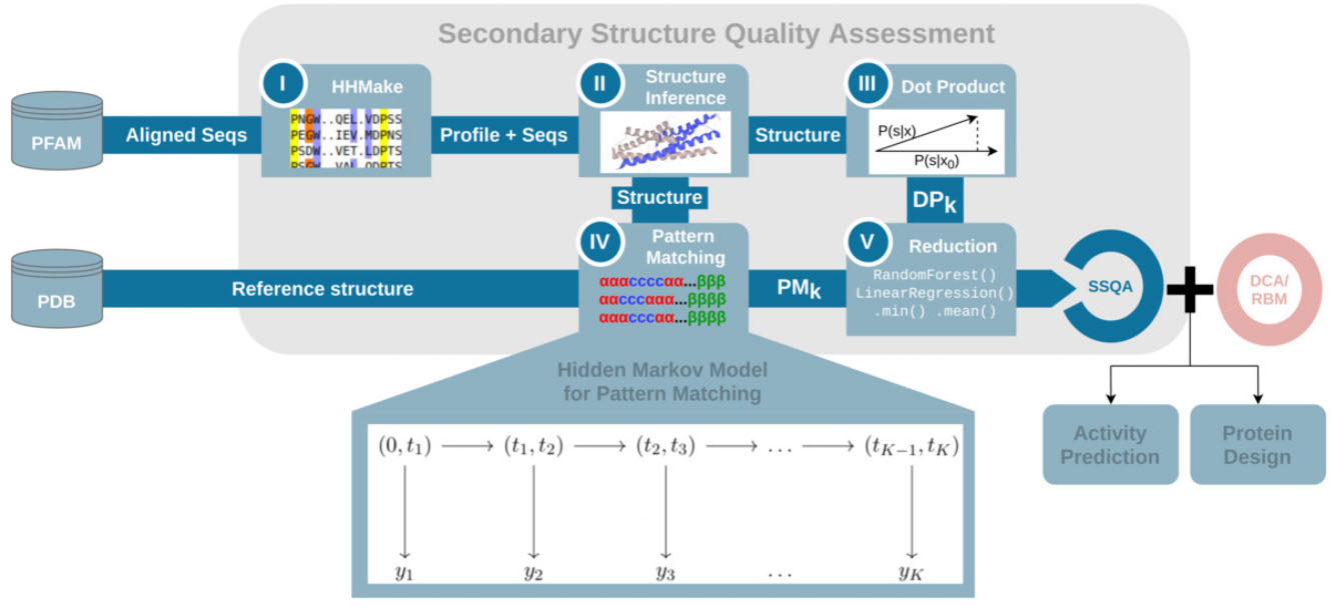
Schema of the SSQA pipeline. First step (I) being the alignment of sequences and computations of the profiles through HHmake (image PFAM), then the structure inference (II, see Section 2.2) through a secondary structure predictor (image UniProt). Then the computation of the DP (III, see Section 3.2) and PM (IV, see Section 3.3) features with reference structure from PDB and finally the reduction (V, see Section 3.4) through unsupervised or supervised reduction

We develop the framework PM to compare the output of the secondary structure predictor and a pattern, which we define as a determined finite sequence of secondary structure elements (3 class or 8 class, see Section 2.2) of undetermined length and position in the amino-acid sequence. Concretely, the protein sequences following the same pattern have the same succession of structure elements but these elements can vary in their lengths and in the positions they correspond to on the amino-acid sequences (see Fig. 2, IV for a visual example). Formally, a pattern *r* is defined as an ordered set of elements called motifs: $r={\left(C_{k}\right)}_{k\in [1,K]}$ where *C_k_* is a secondary-structure class (α-helix, β-strand or coil for 3-state classification).

We want to assess whether the predicted structure $s={\left(s_{i}\right)}_{i\in [1,n]}$ of a sequence follows the expected pattern *r*. We define ${\left(t_{k}\right)}_{k\in [0,K]}$ where *t_k_* represent the position of the end of the motif *C_k_* (defining the transition from motif *C_k_* to motif $C_{k+1}$ for k∈[1,K−1] or the beginning of the motif *C*_1_ for *k* = 0 and the end of the last motif *C_K_* for *k* = *K*). A structure $s\in {\left\{\alpha -\text{helix},\beta -\text{strand},\text{coil}\right\}}^{n}$ is said to match the pattern *r* if there exists ${\left(t_{k}\right)}_{k\in [0,K]}$ such that

1. The pattern covers the whole sequence, i.e. $t_{0}=0$, *t_K_* = *n*.
2. Each motif of the pattern exists: $\forall k,(t_{k+1}-t_{k})\geq 1$.
3. Each motif covers the expected secondary structure: for *i* such as $t_{k-1}\leq i<t_{k}$, we have *s_i_* = *C_k_*.

We hereafter denote by *R* the set of secondary structures *s* that match pattern *r*. We will define Match(x,r) the probability of an amino-acid sequence *x* having a structure that matches *r*:

(10) $$\text{Match}(x,r)=\sum_{s\in R}P(s|x).$$

Brute force computation of Match(x,r) is not possible, as the size of *R* grows exponentially with the length *n* of the sequence. However, it can be calculated in polynomial time, using the distribution defined in Equation (7).

To do so, we make use of the HMM framework. A HMM is a statistical model in which a system follows or is modeled as a Markov process over a set of hidden (not observable) states. Here, these hidden states are the stretches of motifs, $T={\left((t_{k-1},t_{k})\right)}_{k}$. In addition to this Markov process, there is another process generating observable symbols $Y={\left(y_{k}\right)}_{k}$, where the probability of *y_k_* depends only on $\left(t_{k-1},t_{k}\right)$.

We consider the following Markov model:

**Hidden states**: $\left(t_{k-1},t_{k}\right)$, where $t_{k-1}$ and *t_k_* are such that the motif *r_k_* of class *C_k_* is in the interval of sites $\left[t_{k-1},t_{k}-1\right]$.

**Observation states**: $y_{k}\in \{0,1\}$ where *y_k_* = 1 if the motif of index *k* verifies the third matching condition and *y_k_* = 0 otherwise. Given *t_k_*, $t_{k-1}$, *C_k_* we have *y_k_* = 1 with probability $p=\prod_{i\in [t_{k-1},t_{k}-1]}p(s_{i}=C_{k}|x)$.

In the context of this Markov process, *x* has a structure matching pattern *r* if $\forall k\;y_{k}=1$. The computation of Match(x,r) can then be done through the sum-product algorithm (Kschischang *et al.*, 2001), a dynamic programming method for marginalizing the hidden states running in quadratic time and similar to the celebrated Viterbi algorithm. More details about the method are available in Supplementary Section S1. We then obtain Match(x,r) and, for each *k*, $P(t_{k}|x,r)$ and $P(t_{k},t_{k+1}|x,r)$. We define, for each *k*, the length of the motif *r_k_* in the secondary structure, $l_{k}=t_{k}-t_{k-1}$, and compute its probability

(11) $$p(l_{k}=l|x,r)=\sum_{t_{k-1}\leq n-l}P(t_{k-1},t_{k}=t_{k-1}+l|x,r).$$

From this we compute our local PM features, which are the expected lengths of secondary structure motifs

(12) $${\text{PM}}_{k}(x)=\sum_{l_{k}}l_{k}\;p(l_{k}|x,r)\;.$$

### 3.4 Reduction and full pipeline

We then reduce the bag of features (issued from DP or PM) into a single score able to quantify the quality of the secondary structure. Two approaches are possible, depending on the availability of annotated data.

The first method relies on supervised learning. If experimental measures of the goodness (fitness) of proteins are available, working with PM and/or DP features, it is possible to feed these features into a classifier or a regressor for learning with the experimental measures as target. The classifier/regressor used should be adapted to the size of the dataset available. Experimentally, we saw that a Logistic/Linear Regression already performs well but for big enough dataset a Random Forest with 200 trees (from scikit-learn library, Pedregosa *et al.*, 2011) yielded better results even though we noticed over-fitting on the training set (see Section 4.1 and Supplementary Section S3 for more details).

If experimental data are not available we have to rely on ‘unsupervised’ methods. We compute a single score DP from DP features, $\text{DP}(x)=\min_{i}{\text{DP}}_{i}(x)$ or $\text{DP}(x)=\sum_{i}{\text{DP}}_{i}(x)$, and for PM, $\text{PM}(x)=\min_{k}{\text{PM}}_{k}(x)$ or $\text{PM}(x)=\sum_{k}{\text{PM}}_{k}(x)$. It is possible to linearly combine both DP and PM into a single score. We empirically see when optimization was possible that SSQA(x)=DP(x)+PM(x) is usually close to be the optimal linear combination of the score.

After reduction, the pipeline is complete, and we obtain a numerical estimate of the quality of the secondary structure of a given sequence. An overview of this pipeline is shown in Figure 2. Steps II–III–IV (structure inference, PM and DP features) have been developed using PyTorch (Paszke *et al.*, 2019), while step I relies on HHsuite (Steinegger *et al.*, 2019) and step V (reduction) on scikit-learn (Pedregosa *et al.*, 2011) for supervision. PDB structure extraction (Burley *et al.*, 2019) is done with Biotite API (Kunzmann and Hamacher, 2018) in provided repository.

## 4 Results

We assess the performance of our approach on existing datasets of various nature. Russ *et al.* (2020) designed new protein sequences from the DCA model learned from homologous sequences of the Chorismate Mutase (CM) enzyme (PF07736 in PFAM, alignment referred to as NAT), and measured their fitnesses *in vivo*, see Section 4.1. In Section 4.2, we test our approach on 23 mutational effect datasets compiled in Hopf *et al.* (2017) (complete list of reference is available in Supplementary Section S6). Finally, in Section 4.3, we propose an approach to combine SSQA and DCA models in a protein design process and showed potential improvement in the average functionality of the sequences generated.

### 4.1 *A posteriori* screening of DCA-based designed proteins with SSQA

CM is an enzyme that catalyzes an intermediate reaction in the synthesis of aromatic amino acids. Its role in maintaining the balance of these amino acids in the cell is vital, making easy the evaluation of its functionality. In Russ *et al.* (2020), putative protein sequences sampled from DCA model distribution (1), were inserted in *Escherichia coli*, in which the CM gene had been removed. The growth rate of these *E.coli* presented a bimodal distribution that allowed for splitting the sequence dataset into *inactive* and *active* samples.

**Fig. 3.**
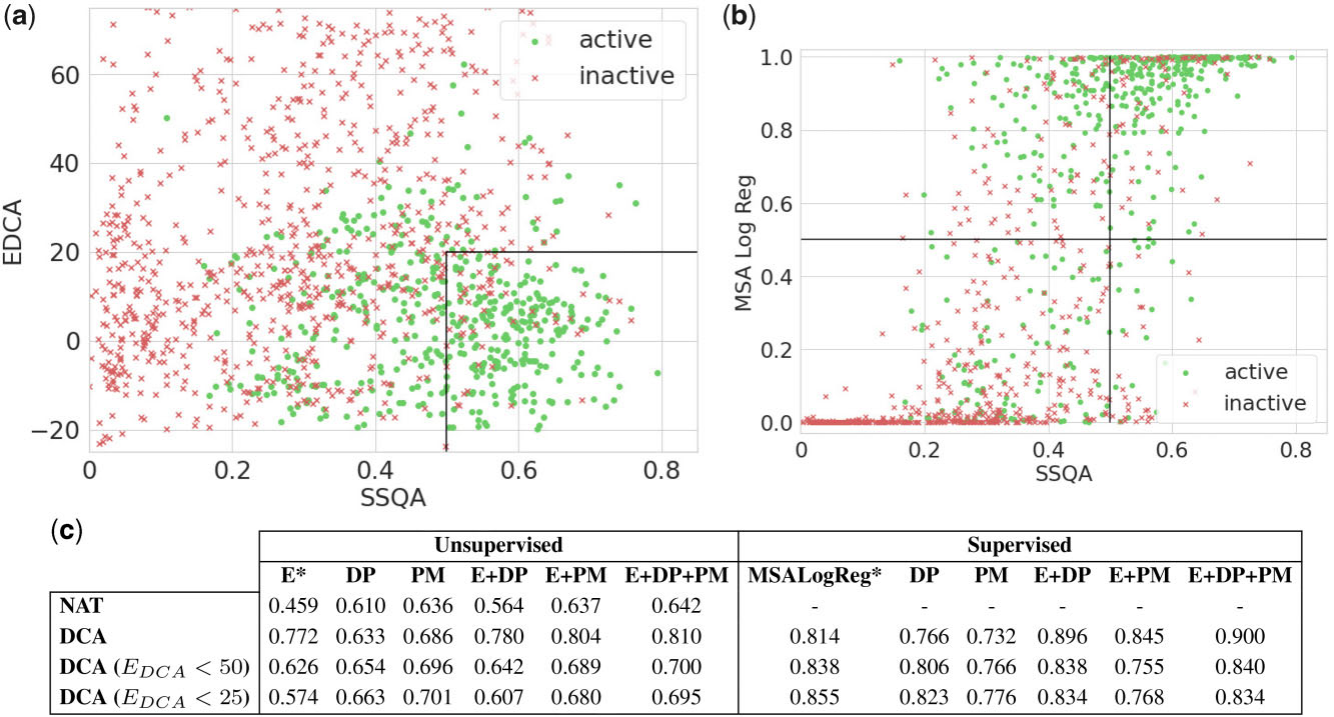
(**a**) *E*_DCA_ and SSQA of generated samples. Green dots are the active samples. As we can see, the combination of DCA energy and SSQA allow a good discrimination. About 82% of the samples in the black block are active. (**b**) MSA Log Reg from Russ *et al.* (2020) and SSQA of generated samples. Green dots are the active samples. MSA Log Reg is performing Logistic Regression of the MSA of the sequences with activity as a target. The Spearman correlation between the two scores is *ρ* = 0.65. (**c**) AUROC for inactive samples detection for different combination of features, MSA Log Reg and *E*_DCA_ from Russ *et al.* (2020) dataset, PM features and DP features with and without supervision. We computed the AUC with the activity as a target for different datasets: natural sequences (NAT), generated sequences (DCA) and subset of the generated sequences by focusing on low-energy samples ($E_{\mathrm{DCA}}<25$) or on samples generated with different sampling temperatures *T* (the higher *T*, the more the samples will be generated with freedom toward the training set). As we can see the use of SSQA features (DP, PM) has a particular interest on low-energy samples that are indistinguishable from natural sequence from statistics of order 1 and 2. The combination DCA energy and SSQA features often yield a very good discrimination of low activity samples (AUC: 0.914 with supervision and 0.810 without on the full dataset)

Our objective is to help discriminating active and inactive protein sequences. We will take a look at several scores. First we consider the DCA energy,$E_{\mathrm{DCA}}(x)$ in (2) (as available in Russ *et al.* (2020) dataset), which corresponds to the negative log-probability (up to an additive constant) in the DCA model. The vast majority of high-energy sequences are inactive, while a substantial fraction of low-energy sequences is active. However, DCA energy alone does not allow for separating active from inactive sequences below some energy threshold, see for instance *E*_DCA_ < 25 in Figure 3c. Russ *et al.* (2020) showed that discrimination performance could be enhanced by a Logistic Regression trained on aligned sequences (MSA) with activity as a target (MSA Log Reg). This method could identify generated sequences similar to the one of the test organism (*E.coli*) in the low-dimensional space spanned by the top components of the sequence data covariation matrix.

We next study the capability of SSQA to discriminate between active and inactive sequences, in particular at low *E*_DCA_. To do so we rely on supervised and unsupervised scoring methods based on the DP and the PM features. Our training set is made of the natural sequences (NAT), whose activities have been determined experimentally (see above). Sequences generated with DCA models in Russ *et al.* (2020) (DCA) will constitute our testing set. Their activity was also experimentally assessed. Taking for *x*_0_ the CM sequence of *E.coli* and its secondary structure in PDB (PDB: *1ECM*) as references, we compute the DP and PM features with both 3- and 8-class secondary structures. For the unsupervised scoring functions, we found that $\text{DP}(x)=\sum_{i}{\text{DP}}_{i}{\left(x\right)}^{\frac{1}{2}}$ and $\text{PM}(x)=\sum_{k}{\text{PM}}_{k}(x)$ yield the most encouraging results (see Figure 3c). For the supervised scoring functions, we train a model through a Random Forest with 200 trees on the natural sequences (NAT) to target the activity of a sequence and evaluated it on generated sequences (DCA). The model was chosen from cross-validation on the training set as describe in Supplementary Section S3.

In Figure 3a, we plot the DCA energy and the supervised SSQA score (DP + PM) on a same graph with active samples in green and inactive ones in red. We see that SSQA helps discriminate active and inactive samples with low energy. Most of the sequences with low energy have been correctly labeled as inactive by SSQA, while most samples with good SSQA and low DCA energy are active. In the low *E*_DCA_-high SSQA domain delimited by the black lines in the figure 85% of the sequences are active. In Figure 3b, we show the scatter plot of MSA Log Reg and of the supervised SSQA score (DP + PM). The high value of the Spearman coefficient underlines the correlations between the correlation between the enrichment method developed by Russ *et al.* (2020) and secondary structure features. This correlation may either reflect a causal effect, i.e. preservation of secondary structure is a key ingredient to the functionality of the protein, or simply that the similarities at the secondary structure level are indicative of the phylogenetic similarities in the CM family.

Last of all we notice in Figure 3c that DP clearly outperforms PM when used with supervision, while PM is better without supervision. This is an important remark to take into consideration, since, for the many studies that do not rely on experimental measurements of protein viability, supervised methods to improve the precision of SSQA cannot be used.

### 4.2 SSQA on mutational datasets

We generalize the method on mutational datasets extracted from multiple mutagenesis studies compiled in Hopf *et al.* (2017). Each of these datasets (available in the Git repository in mut.zip) contain sequences with generally one or few mutations around a wild-type sequence, with the experimentally determined values of their *in vitro* or *in vivo* fitnesses. Hopf *et al.* (2017) perform mutational effect predictions through DCA couplings, see (2), that we take as baseline for our own predictor.

**Fig. 4.**
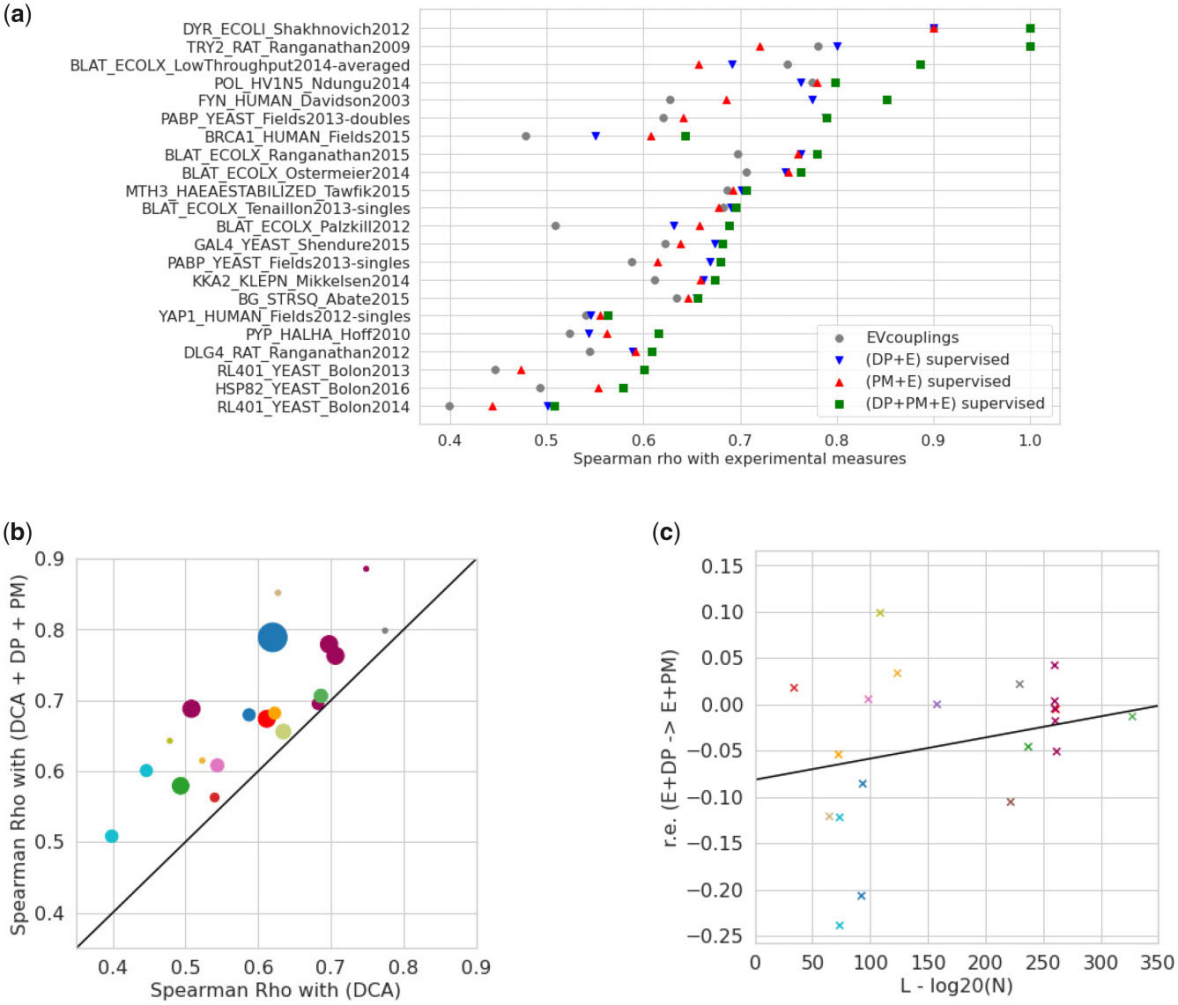
(**a**) Spearman correlations between experimental fitness measurements computed through cross-validation with DCA energy (E) from Hopf *et al.* (2017), DP and PM features, and their combinations, for different datasets. (**b**) Scatter plot of the Spearman correlations *ρ* between the experimental measurements of fitness and the scores combining DCA energy, as well as DP and PM features (E + DP + PM) versus DCA energy only (E) over the different datasets. The sizes of the dots are proportional to the sizes of the corresponding datasets. The improvement brought by SSQA is nonnegligible in particular for bigger datasets. (**c**) Relative enrichment from DP method to PM given the relative size of the dataset *N* compared to the size the sequence L−log 20(N). As the quantity of available annotated data decreases DP become less and less performing compared to PM

To quantify the performance of SSQA, we train models with features computed through DP and PM through cross-validation. The secondary-structure patterns are retrieved from PDB when available, or inferred with the PM inference method. The model we select is a Random Forest Regressor (50 trees) fitted with the experimental fitnesses as targets through cross-validation. We then linearly combine DP and PM scores with the DCA score (energy of mutated sequence) from Hopf *et al.* (2017), weighting of the scores are optimized through Linear Regression and cross-validation. We compute for each obtained score the Spearman correlation *ρ* with the ground-truth experimental measurements. A selection of these correlations can be found in Figure 4a. Figure 4b shows the scatter plot of the Spearman correlations obtained with the DCA coupling estimates (E) only versus the ones where DCA couplings are combined with DP and PM (E + DP + PM). We see that, for most datasets, both DP and PM bring an improvement to the mutational effect prediction.

In Figure 4c, we show the relative improvement $r=\log\frac{\rho (E+\mathrm{PM}+\mathrm{DP})}{\rho (E)}$ as a function of the ratio $L-{\;\text{log}\;}_{20}(N)$, where *L* is the length of the reference sequence and *N* is the size of the dataset. We observe a slight correlation between *r* and $L-{\;\text{log}\;}_{20}(N)$ (Pearson ρ=0.32, *P*-value = 0.14). DP often outperforms PM for large training dataset, whereas PM is superior to DP for small datasets. The few sophisticated features produced by PM are helpful for long proteins or small datasets, but it is advisable to use the many raw features of DP when sequences are short and numerous.

### 4.3 Improved Restricted Boltzmann Machine-based sequence sampling with secondary structure sequence assignment

We now ask whether SSQA can improve graphical-model-based generating models. We consider a RBM energy$E_{\mathrm{RBM}}(x)$ for sequence *x*, and a scoring function *m*(*x*, *r*) of the secondary-structure quality (local or global) of sequence *x* with respect to pattern *r*; *m* can be for instance one of the SSQA metrics introduced above. We propose to sample sequences following the distribution

(13) $$P(x,r)=\frac{1}{Z}\;1_{m(x,r)>\lambda }\;e^{-E_{\mathrm{RBM}}(x)}\;,$$

where *Z* is a normalization constant, and the indicator function 1 rejects all sequences with scores lower than the threshold *λ*.

In practice, we sample the RBM distribution of (4) with Gibbs sampling, see Algorithm 2. Then we perform rejection sampling as following to simulate P(x,r).

Using the CM dataset described in Section 4.1, we train a RBM from the NAT alignment of PFAM (PF07736) and PCD. The ‘$L_{1}b$’ normalization defined in Tubiana *et al.* (2019) is used. The hidden layer is composed of 200 Gaussian units. After training, the RBMs are used to sample 2000 sequences by Gibbs sampling (30 steps). Rejection with different thresholds is performed, to enforce harder and harder secondary structure requirement. We use the unsupervised PM score (AUC: 0.686 in Table 3) to reject samples, as this score requires knowledge about the secondary structure only, thereby making the method applicable to proteins for which no experimental data is available. Newly generated samples are available in pfam/russ/gen_data in the Git repository.

**Fig. 5.**
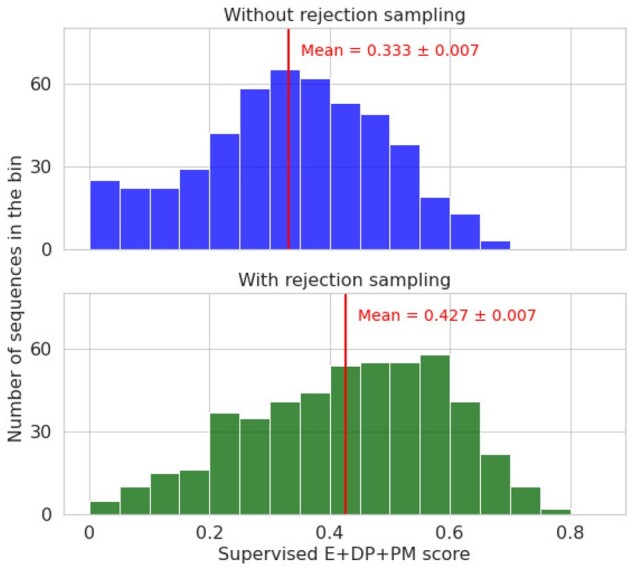
Distribution of probability to be active predicted from Random Forest classifier with supervised E + DP + PM features (Section 4.1), of 500 sequences generated by RBM without and with rejection sampling (rejection unsupervised PM SSQA). The numbers of putatively active sequences are much higher with rejection (green) than without (blue)

For validation of our method, we use the supervised combined E + DP + PM score (AUC: 0.899 in Figure 3c) to assess the quality of our generated samples, even though methods used for rejection and for functionality assessment are similar, the latter still gives a good idea of the improvement brought by the rejection. The supervised E + DP + PM score predict that the fraction of sequences predicted active in the generated dataset are 26% and 51% with, respectively, no (λ=−∞) and high (λ=0.65) rejection. Results are displayed in Figure 5, where a clear shift toward good structures samples when adding rejection based on SSQA (*t*-test gave us a *P*-value *P*$\;=7.3\times 10^{-19}$), which strongly suggest that experimental determination of activity would also lead to an improvement in the share of active sequences designed.

## 5 Conclusion

In summary, we have proposed multiple scoring functions for assessing the compatibility of protein sequences with respect to a reference secondary structure. Our approach is computationally tractable, has intuitive meaning, and shows promising performance. *A posteriori* validation of sequences generated in previous works shows the ability of our method to detect dysfunctional proteins, and constitutes an improvement compared to standard graphical-model-based methods. These results strongly suggest that quality assessment is a practical way to exploit secondary structure, despite the ∼15% error rate of the best available secondary structure prediction algorithms.

The results we report on CMs or on mutational effect datasets showed a great complementarity between SSQA and DCA; for instance, functionality of sequences with both good SSQA and good DCA energy have been shown to be very high (>80% for CMs). It is not surprising that DCA or RBM captures functionally relevant information on residues (for instance, at or close to binding sites) beyond secondary structure alone. However, it is less clear what statistical features of the sequence data are overlooked by graphical models, and, yet, essential to secondary structure prediction. A preliminary answer to this question can be found in Supplementary Section S5. Indeed, for the betalactamase family (see Majiduddin *et al.*, 2002), SSQA brings particular improvement in the activity prediction task for mutations happening on *β*-strand, where DCA models are failing to yield good prediction. While it may not be inconceivable that accounting for some *β*-motifs may require a complex pattern of couplings, beyond what DCA can accommodate for, further systematic studies are required to understand the origin of the complementarity between their scores.

In addition, we observed that SSQA methods, in particular PM, may lead to enhanced performance with little sequence annotation e.g. additional structural information, or little experimental data. Indeed, state-of-the-art secondary structure prediction software have been tested and validated on huge datasets, including all protein families with known structures. Use of these methods for SSQA of sequences attached to a single protein family may therefore be seen as an illustration of knowledge transfer. In this context, addition of tertiary-structure quality assessment method such as in Baldassarre *et al.* (2021) would be interesting for further developments.

Last of all, the efficient computation of SSQA scores reported in this work suggest other applications and their integration at the heart of sequence generative processes, such as sampling with rejection, as done here. Furthermore, it would be interesting to integrate these scores into reward functions for Reinforcement Learning process or loss functions for Neural Networks, in generative networks (such as Hawkins-Hooker *et al.*, 2020; Repecka *et al.*, 2019) or representation network (Alley *et al.*, 2019; Rives *et al.*, 2019), which is made possible by their differentiability.

## Funding

S.C. and R.M. were supported by the Agence Nationale de la Recherche [grant ANR-17-CE30-0021 RBMPro and ANR-19-CE30-0021] Decrypted. C.M. is recipient of a PhD funding from AMX program, Ecole Polytechnique and benefits from financial support from the Centre de Recherche Interdisciplinary (CRI) through ‘Ecole Doctorale Frontières de l’Innovation en Recherche et Education—Programme Bettencourt’. 

*Conflict of Interest*: none declared.

## Supplementary Material

btab442_Supplementary_Data
