# Supplementary material for: Improving sequence-based modeling of protein families using secondary-structure quality assessment
Source: Bioinformatics. 2021 Jun 12;37(22):4083–90. doi: 10.1093/bioinformatics/btab442 (PMC9502231; doi:10.1093/bioinformatics/btab442)
Supplement: btab442_Supplementary_Data [file btab442_supplementary_data.pdf]

Supplementary Material for *Improving sequence-based modeling of protein families using secondary structure quality assessment*

Cyril Malbranke, David Bikard, Simona Cocco and Rémi Monasson

June 3, 2021

Contents

1 Pattern Matching details **2**
1.1 Likelihood of a pattern 2
1.2 Pattern Inference 3

2 Errors done by Secondary Structure Predictor **5**

3 Model Selection for Supervised SSQA with Chorismate Mutases **7**

4 Supplementary figures about *A posteriori screening of DCA-based designed proteins with SSQA* **7**

5 Improvements of SSQA in function of the secondary structure in the betalactamase **10**

6 References to Datasets used for "Secondary structure quality assessment on mutational datasets" **13**

1 Pattern Matching details

A pattern r is defined as an ordered set of elements called **motifs** $(r_k = (C_k, d_k))_{1 \leq k \leq K}$ where r_k is the motif, C_k the motif class (α-helix, β-strand or coil), d_k is a distribution of probability defining the transition matrix.

A structure $s \in \{\alpha\text{-helix}, \beta\text{-strand}, \text{coil}\}^n$ is said to **match** the pattern r if $\exists (t_k)_{k \leq K}$ such as :

1. $t_0 = 0, t_K = n$
2. $\forall k > 0, t_k - t_{k-1} \geq 1$
3. $\forall i$ such as $t_{k-1} \leq i < t_k$, we have $x_i = C_k$

From now on, we define the distribution d_k based on the minimal and maximal length of the different class of secondary structure we observe in available sequences in PDB. Let's consider a class of secondary structure C. We denote by $M(C)$ the maximum length of a structure of class C we observe in PDB and $m(C)$ the minimum length. For each k, we then have d_k define as :

$$d_k(t_{k-1}, t_k) = \frac{\mathbf{1}_{m(C_k) \leq t_k - t_{k-1} \leq M(C_k)}}{M(C_k) - m(C_k) + 1}$$

d_k is then a uniform distribution on the acceptable length of the structure. Other distribution have been tested based on the frequencies of the length of the motifs in PDB, but yielded lower performances.

Afterwards we will denote by $R = \{s \in \mathcal{P}(\{\alpha\text{-helix}, \beta\text{-strand}, \text{coil}\}^n)\}$ the set of secondary structures that match the pattern r. We will define $\text{Match}(x, r)$ the probability of x having a structure that matches r

$$\text{Match}(x, r) = \sum_{s \in R} \mathbb{P}(s|x) \tag{1}$$

We explain below how to compute $\text{Match}(x, r)$ in a time polynomial in the sequence length n.

1.1 Likelihood of a pattern

Given the probability matrix of the structure P^x defined previously we want to assess the probability of this probabilistic structure to match r, denoted by $\text{Match}(x, r)$.

It is possible to represent this problem with a Hidden Markov Model. A **Hidden Markov Model** (HMM) is a statistical model in which a system followed or is modeled as a Markov process $Z = (z_k)_k$, where Z is not observable. In addition of this Markov process, there is another process $Y = (y_k)_k$, observable and such that $\forall k$, y_k depends only on z_k. The objectives we can meet with these model are multiple : decoding (finding the most likely z), marginalizing (finding $p(z_k|y_1, ..., y_k)$) ...

In our case we consider the following Markov Model :

Hidden states : $z_k = (t_{k-1}, t_k)$ the intervals of residues $[t_{k-1}, t_k[$ of the motif r_k of class C_k.

Transition probability : $p(t_k|t_{k-1}) = d_k(t_{k-1}, t_k)$.

Observation states : $y_k \in \{0, 1\}$ where $y_k = 1$ if a motif matching $r_k = (C_k, d_k)$ conditions was emitted. given t_k, t_{k-1}, C_k, we have $y_k \sim q_k = \mathcal{B}(\prod_{i \in [t_{k-1}, t_k[} p(s_i = C_k|x))$ where B is a Bernoulli law

We can see from the previous definition that x as a structure s that matches pattern r if and only if $\forall k, y_k = 1$. We then have :

$$\text{Match}(x, r) = p(y_1 = 1, \ldots, y_K = 1|x, r)$$

In order to compute this probability, we will rely on our Markov chain. We here recall a dynamic programming way of marginalizing a Hidden Markov Model with a process called **sum product algorithm** (see [13]). For a HMM model with observations y_k and hidden states z_k we define recursively :

$$\alpha_{k+1}(z_{k+1}) = p(y_{k+1}|z_{k+1}) \sum_{z_k} p(z_{k+1}|z_k)\alpha_k(z_k)$$
$$\beta_k(z_k) = \sum_{z_{k+1}} p(y_{k+1}|z_{k+1})p(z_{k+1}|z_k)\beta_{k+1}(z_{k+1})$$

we then have after computation :

$$\alpha_k(z_k)\beta_k(z_k) = p(z_k, y_0, \ldots, y_K)$$
$$\sum_{z_k} \alpha_k(z_k)\beta_t(z_k) = p(y_0, \ldots, y_K)$$

We will be using the sum-product algorithm with our own Hidden Markov Model (Figure 1). After re-arrangement, it gives us :

$$\alpha_{k+1}(t_{k+1}) = \sum_{t_k} p(s_{[t_k,t_{k+1}[} = C_{k+1})p(t_{k+1}|t_k)\alpha_k(t_k)$$
$$\beta_k(t_k) = \sum_{t_{k+1}} p(s_{[t_k,t_{k+1}[} = C_{k+1})p(t_{k+1}|t_k)\beta_{k+1}(z_{k+1})$$

In this case, we have $\forall k$:

$$\begin{aligned}\text{Match}(x, r) &= p(y_1 = 1, \ldots, y_N = 1|x, r)\\ &= \sum_{t_k} \alpha_k(t_k)\beta_k(t_k)\end{aligned}$$

And in particular :

$$\begin{aligned}\text{Match}(x, r) &= p(y_1 = 1, \ldots, y_K = 1|x, r)\\ &= \alpha_K(n)\beta_K(n) = \alpha_K(n)\end{aligned}$$

1.2 Pattern Inference

For a lot of proteins (in particular the ones that are driving attention), structures from which we infer patterns are available online, we retrieve most of them on the Protein Data Bank [3]. For others we may be require to infer the pattern from the result of our secondary structure prediction.

From now on, we define the distribution d_k based on the distribution of length of the different class of secondary structure we observe in available sequences in PDB. Let us consider a class of secondary structure C. We denote by $f(C, l)$ the frequency of the length l for the structure of class C we observe in PDB. For each k, we then have d_k defined as :

$$d_k(C_k, t_{k-1}, t_k) = f(C_k, t_k - t_{k-1})$$

We now expand our Hidden Markov Model so the class of each motif is integrated in the hidden state. We will have to also adapt our transition probability to our new model (see Figure 2). The adapted model is then defined as follows:

Hidden states : $z_k = (C_k, t_{k-1}, t_k)$ the class C_k of the motif r_k the intervals of residues $[t_{k-1}, t_k[$ of the motif r_k of class C_k.

Transition probability : The transition probability will be modelled as
$p(C_k, t_{k-1}, t_k | C_{k-1}, t_{k-1}, t_{k-2}) = p(C_k | C_{k-1}) \cdot d_k(C_k, t_{k-1}, t_k)$

where $p(C_k | C_{k-1})$ is the probability of having a motif of class C_k following a motif of class C_{k-1}, and d_k the distribution of probability define above.

From a technical point of view, since we do not a priori know the length of the motif, it is necessary to add a final stationary state S, with $p(C_k = S | C_{k-1} = S) = 1$ and $d(S, t_{k-1}, t_k) = \mathbf{1}_{t_{k-1}=t_k}$.

With this formalism we can run the max-product algorithm (or Viterbi algorithm) to find the most likely pattern given the predicted secondary structure.

2 Errors done by Secondary Structure Predictor

We noticed two kinds of errors done by the algorithm, against which Pattern Matching is robust :

- **Border errors**: A lot of errors are done at the transition between two structures because of uncertainty of where a structure begin and where the other end. Though these errors are numerous as we can see on Figure 3, they do not affect much the performance of Pattern Matching as the length of the structure is flexible in the pattern.
- **Weak errors**: When the structure is incorrectly predicted on a residue, the true label often has a non-negligible likelihood, i.e. above 0.1 in more than 60% (see Figure 4) of the cases. Pattern Matching is robust against these errors as it can correct them with a low cost for the Match score.

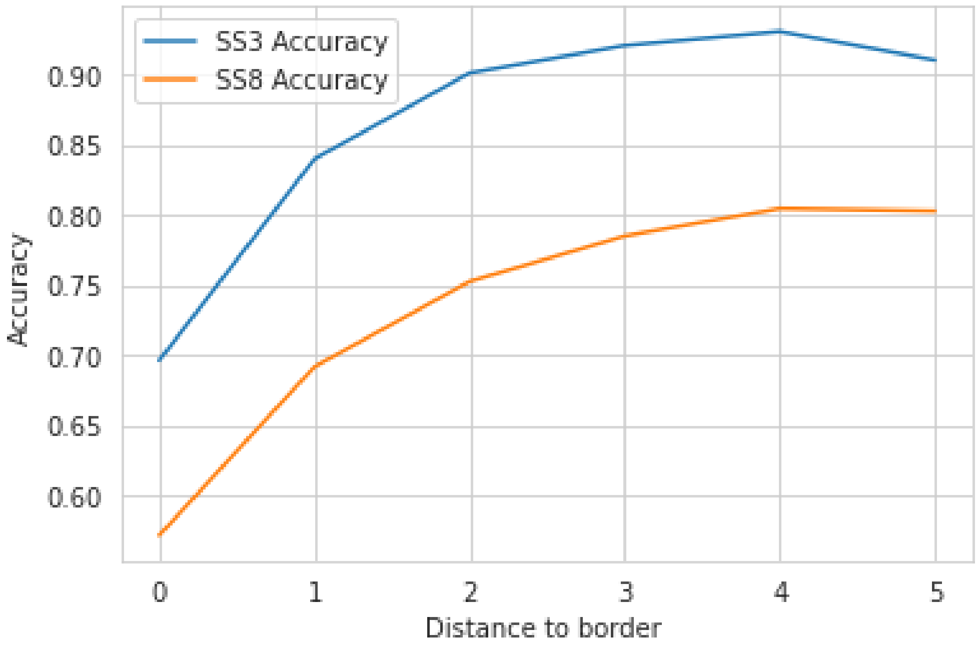

Figure 3: Accuracy (share of the labels predicted correctly) as a function of the distance to a transition. Accuracy drops at the boundary between two structures.

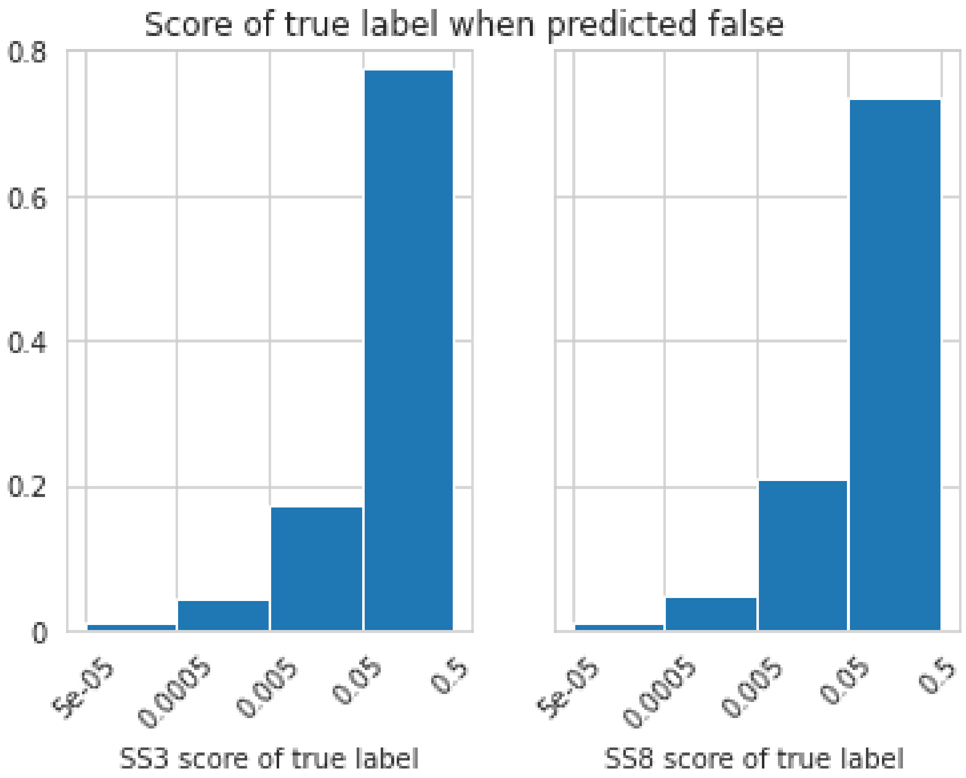

Figure 4: Likelihood of the true secondary structure when a wrong structure is predicted. To remove the bias created by border errors we focused on residues at a distance at least 2 from the boundary between two structures. Most true labels still have pretty high accuracy.

3 Model Selection for Supervised SSQA with Chorismate Mutases

We worked with different models with Scikit Learn [19] for supervised reduction of Pattern Matching and Dot Product with SSQA. Here we display the AUCs obtained with different models: sklearn.linear_model.LogisticRegression(), sklearn.ensemble.GradientBoostingClassifier(), sklearn.ensemble.RandomForestClassifier({10, 50, 200}).

To select the model we worked only with the training set of natural sequences. We proposed two methods to evaluate the models :

- **Method 1** : We extract a **random** sample of 20% of the training dataset for validation. We then train the models with the training dataset and evaluate them (by computing the AUC) on the validation set. We do this operation 100 times to lower the variance.
- **Method 2** : We build 70% **similarity clusters** of similar sequences and we pick at random clusters representing around 20% of the training set for validation. We then train the models with the training dataset and evaluate them (by computing the AUC) on the validation set. We do this operation 100 times to lower variance

Overall we see that Logistic Regression performs slightly worst or as well as Random Forest with 200 trees when it comes to predict activity for samples different from the ones in the training set (with validation set built from similarity clusters). Random Forest though is overfitting leading to better performance with random validation samples susceptible to be close from the one in the training set.

		**Random**		**Similarity Clusters**	
	**n**	**Train**	**Val**	**Train**	**Val**
**Logistic Regression**	-	0.815	0.752	0.819	**0.725**
**Gradient Boosting**	-	0.999	0.757	0.998	0.727
**Random Forest**	10	1.000	0.733	1.000	0.698
**Random Forest**	50	1.000	0.764	1.000	0.723
**Random Forest**	200	1.000	**0.772**	1.000	**0.732**

4 Supplementary figures about *A posteriori screening of DCA-based designed proteins with SSQA*

In Figure 5, we plot the ROC curve for both unsupervised and supervised metrics. Unsupervised scores, though they perform less well than supervised scores, are able to achieve some discrimination of active and inactive samples, potentially helping improving sequence generation.

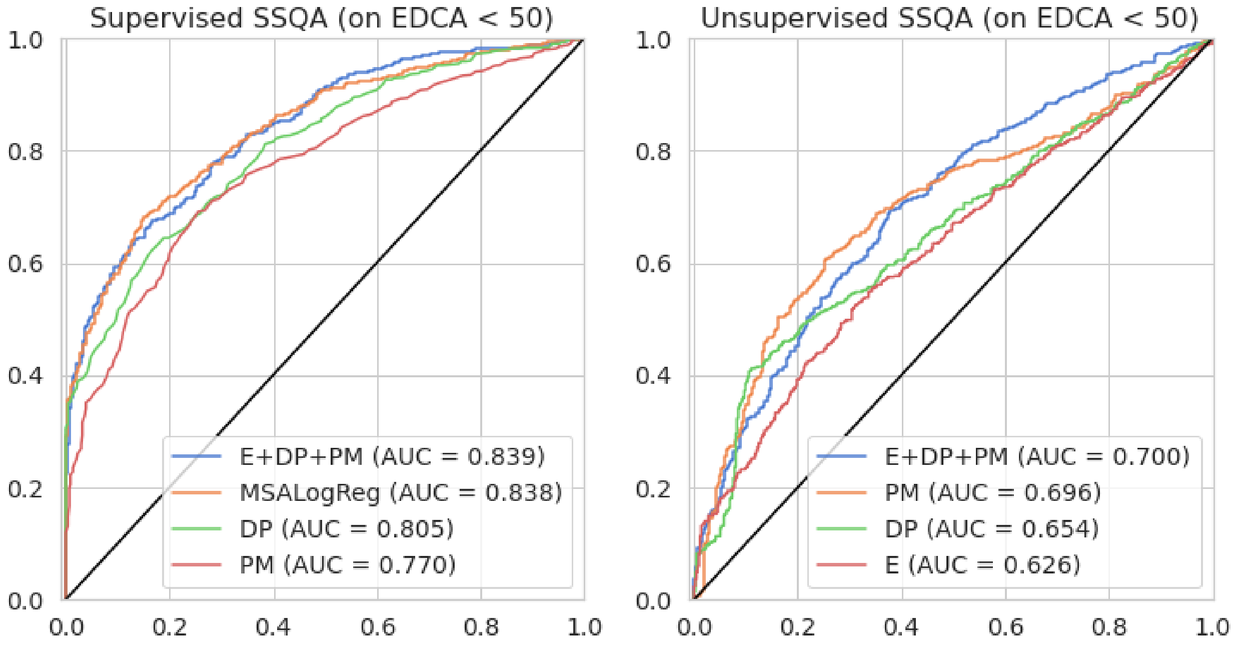

Figure 5: ROC Curve for inactive samples detection with unsupervised and supervised SSQA scores. We focused on low energy samples statistically equivalent to natural samples in terms of statistics of orders 1 and 2.

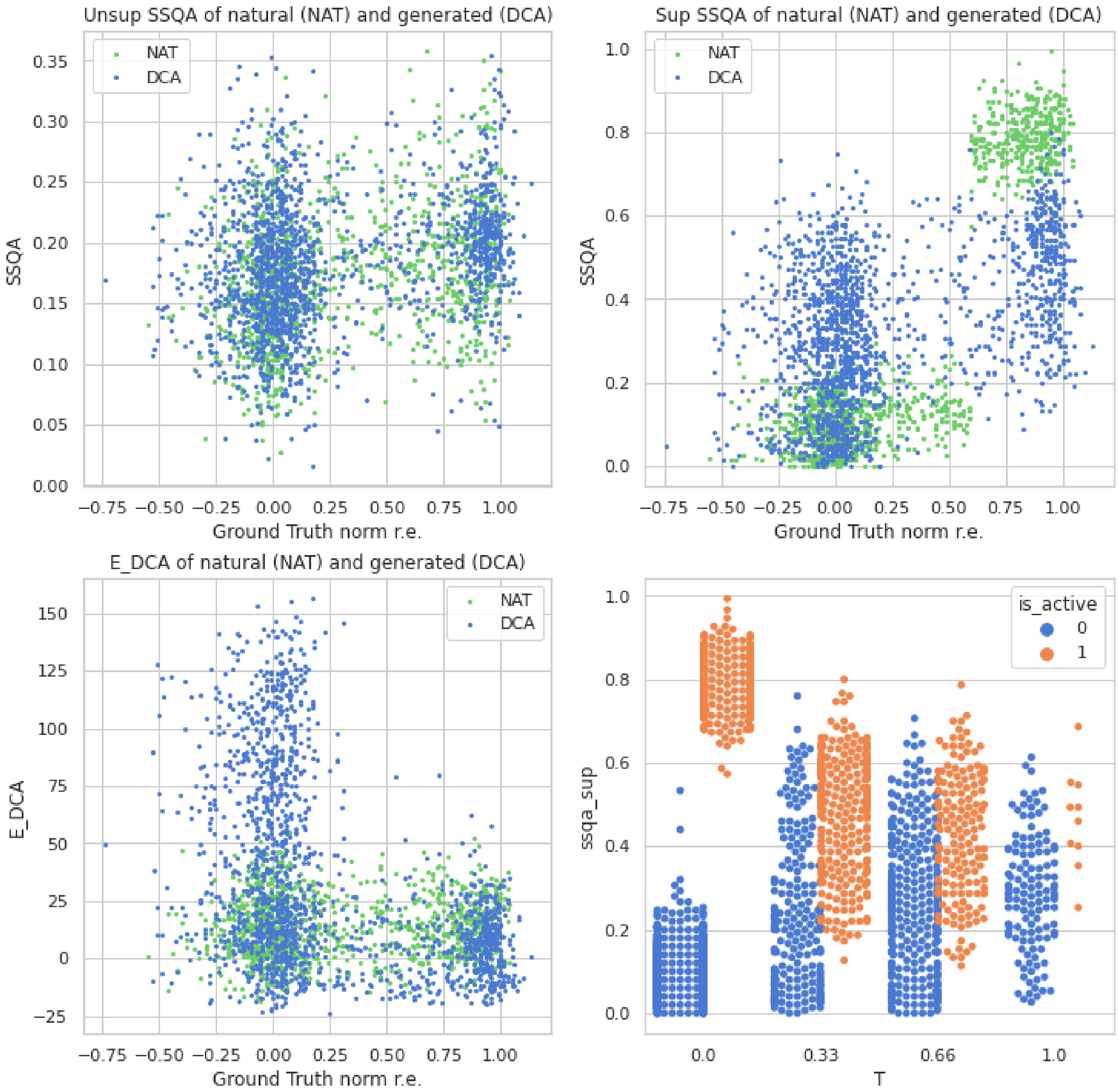

Figure 6: Plot of *unsupervised* SSQA, *supervised* SSQA and E_{DCA} of generated and natural sequences in function of the exprimental activity (Around 0 being inactive, around 1 being active). DCA is able to discriminate easily a lot of bad sequences but fails for some of them. *Supervised* SSQA is also very able to discriminate some bad sequences. Unsupervised SSQA is also able to discriminate inactive sequences though it performs less well than Supervised SSQA. Last panel: violinplot as a function of the temperature of generation (0 corresponds to Natural Sequences). *supervised* SSQA shows a good discrimination at every level of temperature.

5 Improvements of SSQA in function of the secondary structure in the betalactamase

We considered single mutations dataset sequence from beta lactamase [4] (Uniprot ID : P62593). Activities for each single mutation had been experimentally determined (see Figure 7) and the experimentally determined secondary structure is also available (PDB Id : 4ZJ1). We linearly combined Dot Product and Pattern Matching features we computed with taking DCA energy from [10], and built activity predictors out of these metrics. We below computed for mutations happening on each kind of structure the balanced accuracy of the activity prediction task.

Balanced accuracy differs from usual accuracy in which it mean the accuracy on positive and negative label, therefore being able to better evaluate balanced dataset. With TP (number of true positives), TN (true negative), FN (false negative) and FP (false positive), we have the following formula for balanced accuracy :

$$\texttt{balanced-accuracy} = \frac{1}{2}\left(\frac{TP}{TP+FN} + \frac{TN}{TN+FP}\right) \tag{2}$$

In comparison, usual accuracy can be expressed as :

$$\texttt{accuracy} = \frac{TP+TN}{TP+FN+TN+FP} \tag{3}$$

In Figure 8 (with 3-class structure) or Figure 9 (with 8-class structure), predictor based on DCA energy was able to reach 70% to 75% accuracy on α-helix and coil but failed on β-strand. SSQA predictor brought significant improvements in particular on β-strand.

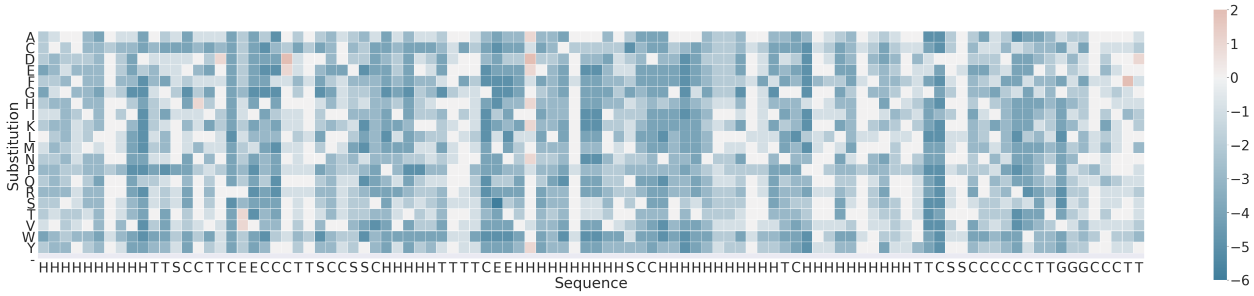

Figure 7: Experimental Mutation Effects on a segment of the beta-lactamase (structure in x-axis). Red and blue squares show, respectively, increasing and decreasing activities.

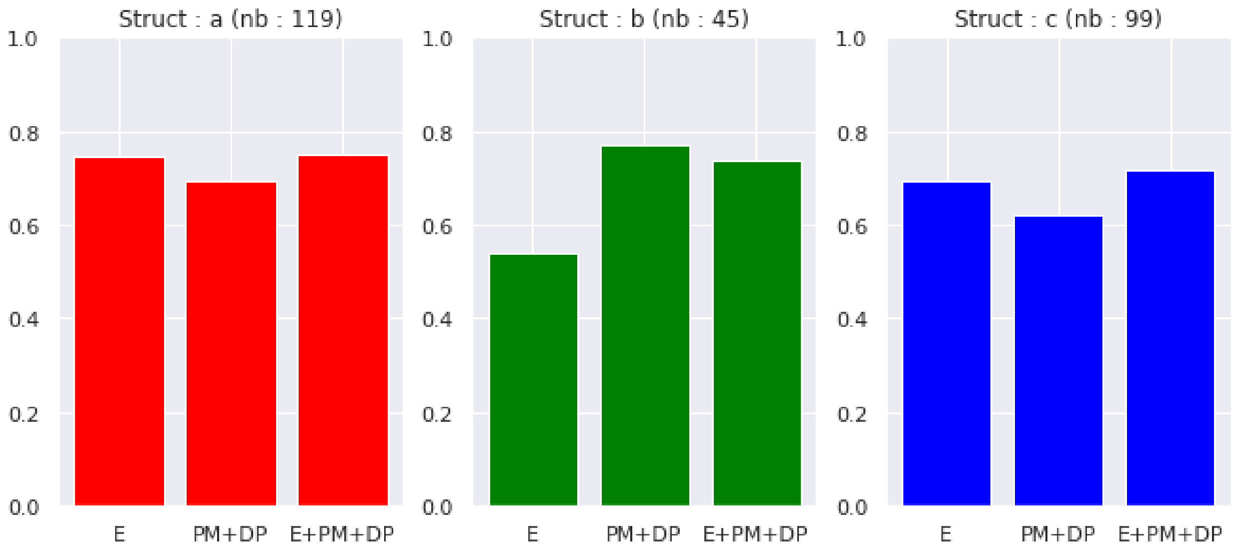

Figure 8: Balanced accuracy for activity prediction on Beta-lactamase for single mutations given the 3-class secondary structure of the mutated residue. SSQA brings particular improvement on β-strands where interaction between residues are usually more complex than in α-helix.

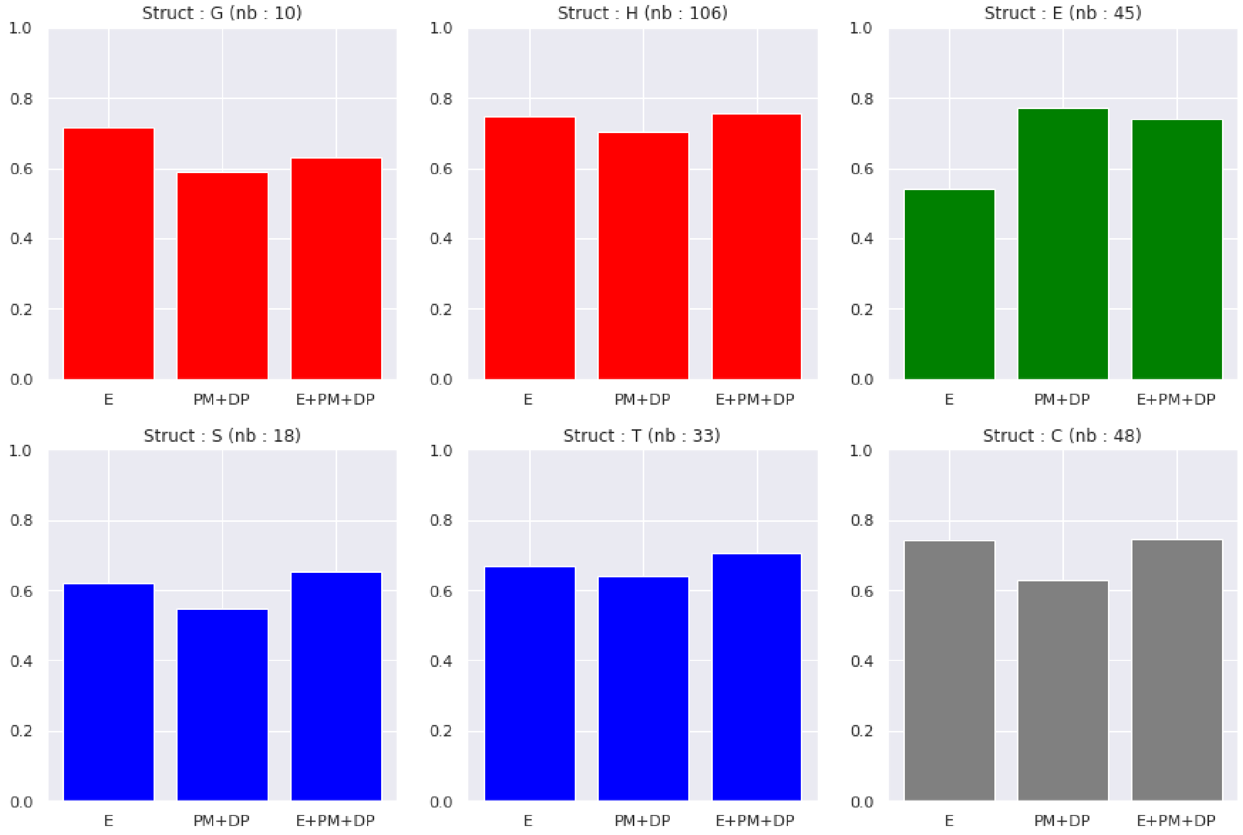

Figure 9: Balanced accuracy for activity prediction on Beta-lactamase for single mutations given the 8-class secondary structure (only 6 classes are present in the structure) of the mutated residue. SSQA brings particular improvement on β-strands where interaction between residues are usually more complex than in α-helix.

6 References to Datasets used for ”Secondary structure quality assessment on mutational datasets”

Here is a list of dataset collected in Hopf et al. [10] and used in section 4. :

ID	Reference
POLG_HCVJF_Sun2014	Qi et al., PLOS Pathogens 2014 [21]
UBE4B_MOUSE_Klevit2013-singles	Starita et al., PNAS 2013 [26]
PA_FLU_Sun2015	Wu et al., PLOS Genetics [29]
RL401_YEAST_Bolon2014	Roscoe et al., JMB 2014 [24]
PABP_YEAST_Fields2013-singles	Melamed et al., RNA 2013 [16]
GAL4_YEAST_Shendure2015	Kitzmann et al., Nat Methods 2015 [12]
RL401_YEAST_Bolon2013	Roscoe et al., JMB 2013 [25]
PABP_YEAST_Fields2013-doubles	Melamed et al., RNA 2013 [16]
HG_FLU_Bloom2016	Doud & Bloom, Viruses 2016 [7]
DLG4_RAT_Ranganathan2012	McLaughlin et al., Nature 2012 [15]
BG_STRSQ_Abate2015	Romero et al., PNAS 2015 [23]
BLAT_ECOLX_Palzkill2012	Deng et al., JMB 2012 [5]
BLAT_ECOLX_Ostermeier2014	Firnberg et al., Mol Biol Evol 2014 [8]
HSP82_YEAST_Bolon2016	Mishra et al., Cell Reports 2016 (in press) [18]
BLAT_ECOLX_Ranganathan2015	Stiffler et al., Cell 2015 (Table S1 and S4) [28]
BRCA1_HUMAN_Fields2015	Starita et al., Genetics 2015 (Table S2) [27]
KKA2_KLEPN_Mikkelsen2014	Melnikov et al., NAR 2014 [17]
YAP1_HUMAN_Fields2012-singles	Araya et al., PNAS 2012 [1]
MTH3_HAEAESTABILIZED_Tawfik2015	Rockah-Shmuel et al., PLOS Comp Bio 2015 (File S3) [22]
PYP_HALHA_Hoff2010	Philip et al., PNAS 2010 (Table S1) [20]
BLAT_ECOLX_LowThroughput2014-averaged	Firnberg et al., Mol Biol Evol 2014 [8]
FYN_HUMAN_Davidson2003	Di Nardo et al., JMB 2003 (Table 2 and 3) [6]
DYR_ECOLI_Shakhnovich2012	Bershtein et al., PNAS 2012 (Tables 1, S1, S2) [2]
POL_HV1N5_Ndungu2014	Mann et al., PLOS Comp Bio 2014 (Supplementary Table 1, 2) [14]
TRY2_RAT_Ranganathan2009	Halabi et al., Cell 2009 (Table S2) [9]
BLAT_ECOLX_Tenaillon2013-singles	Jacquier et al., PNAS 2013 (Supplementary Data 1) [11]

References

[1] Carlos L. Araya, Douglas M. Fowler, Wentao Chen, Ike Muniez, Jeffery W. Kelly, and Stanley Fields. A fundamental protein property, thermodynamic stability, revealed solely from large-scale measurements of protein function. *PNAS*, 109(42):16858–16863, October 2012. Publisher: National Academy of Sciences Section: Biological Sciences.

[2] Shimon Bershtein, Wanmeng Mu, and Eugene I. Shakhnovich. Soluble oligomerization provides a beneficial fitness effect on destabilizing mutations. *PNAS*, 109(13):4857–4862, March 2012. ISBN: 9781118157107 Publisher: National Academy of Sciences Section: Biological Sciences.

[3] Stephen K Burley, Helen M Berman, Charmi Bhikadiya, Chunxiao Bi, Li Chen, Luigi Di Costanzo, Cole Christie, Ken Dalenberg, Jose M Duarte, Shuchismita Dutta, Zukang Feng, Sutapa Ghosh, David S Goodsell, Rachel K Green, Vladimir Guranović, Dmytro Guzenko, Brian P Hudson, Tara Kalro, Yuhe Liang, Robert Lowe, Harry Namkoong, Ezra Peisach, Irina Periskova, Andreas Prlić, Chris Randle, Alexander Rose, Peter Rose, Raul Sala, Monica Sekharan, Chenghua Shao, Lihua Tan, Yi-Ping Tao, Yana Valasatava, Maria Voigt, John Westbrook, Jesse Woo, Huanwang Yang, Jasmine Young, Marina Zhuravleva, and Christine Zardecki. RCSB Protein Data Bank: biological macromolecular structures enabling research and education in fundamental biology, biomedicine, biotechnology and energy. *Nucleic Acids Research*, 47(D1):D464–D474, January 2019.

[4] Zhifeng Deng, Wanzhi Huang, Erol Bakkalbasi, Nicholas G. Brown, Carolyn J. Adamski, Kacie Rice, Donna Muzny, Richard A. Gibbs, and Timothy Palzkill. Deep sequencing of systematic combinatorial libraries reveals -lactamase sequence constraints at high resolution. *Journal of Molecular Biology*, 424(3):150 – 167, 2012.

[5] Zhifeng Deng, Wanzhi Huang, Erol Bakkalbasi, Nicholas G. Brown, Carolyn J. Adamski, Kacie Rice, Donna Muzny, Richard A. Gibbs, and Timothy Palzkill. Deep Sequencing of Systematic Combinatorial Libraries Reveals -Lactamase Sequence Constraints at High Resolution. *Journal of Molecular Biology*, 424(3):150–167, December 2012.

[6] Ariel A. Di Nardo, Stefan M. Larson, and Alan R. Davidson. The relationship between conservation, thermodynamic stability, and function in the SH3 domain hydrophobic core. *J Mol Biol*, 333(3):641–655, October 2003.

[7] Michael B. Doud and Jesse D. Bloom. Accurate Measurement of the Effects of All Amino-Acid Mutations on Influenza Hemagglutinin. *Viruses*, 8(6), June 2016.

[8] Elad Firnberg, Jason W. Labonte, Jeffrey J. Gray, and Marc Ostermeier. A Comprehensive, High-Resolution Map of a Gene's Fitness Landscape. *Molecular Biology and Evolution*, 31(6):1581–1592, June 2014.

[9] Najeeb Halabi, Olivier Rivoire, Stanislas Leibler, and Rama Ranganathan. Protein Sectors: Evolutionary Units of Three-Dimensional Structure. *Cell*, 138(4):774–786, August 2009. Publisher: Elsevier.

[10] Thomas Hopf, John Ingraham, Frank Poelwijk, Charlotta Schärfe, Michael Springer, Chris Sander, and Debora Marks. Mutation effects predicted from sequence co-variation. *Nature Biotechnology*, 35, January 2017.

[11] Hervé Jacquier, André Birgy, Hervé Le Nagard, Yves Mechulam, Emmanuelle Schmitt, Jérémy Glodt, Beatrice Bercot, Emmanuelle Petit, Julie Poulain, Guilène Barnaud, Pierre-Alexis Gros, and Olivier Tenaillon. Capturing the mutational landscape of the beta-lactamase TEM-1. *PNAS*, 110(32):13067–13072, August 2013. Publisher: National Academy of Sciences Section: Biological Sciences.

[12] Jacob O. Kitzman, Lea M. Starita, Russell S. Lo, Stanley Fields, and Jay Shendure. Massively parallel single-amino-acid mutagenesis. *Nature Methods*, 12(3):203–206, March 2015. Number: 3 Publisher: Nature Publishing Group.

[13] F.R. Kschischang, B.J. Frey, and H.-A. Loeliger. Factor graphs and the sum-product algorithm. *IEEE Transactions on Information Theory*, 47(2):498–519, February 2001. Conference Name: IEEE Transactions on Information Theory.

[14] Jaclyn K. Mann, John P. Barton, Andrew L. Ferguson, Saleha Omarjee, Bruce D. Walker, Arup Chakraborty, and Thumbi Ndung'u. The fitness landscape of HIV-1 gag: advanced modeling approaches and validation of model predictions by in vitro testing. *PLoS Comput Biol*, 10(8):e1003776, August 2014.

[15] Richard N. McLaughlin Jr, Frank J. Poelwijk, Arjun Raman, Walraj S. Gosal, and Rama Ranganathan. The spatial architecture of protein function and adaptation. *Nature*, 491(7422):138–142, November 2012. Number: 7422 Publisher: Nature Publishing Group.

[16] Daniel Melamed, David L. Young, Caitlin E. Gamble, Christina R. Miller, and Stanley Fields. Deep mutational scanning of an RRM domain of the Saccharomyces cerevisiae poly(A)-binding protein. *RNA*, 19(11):1537–1551, January 2013. Company: Cold Spring Harbor Laboratory Press Distributor: Cold Spring Harbor Laboratory Press Institution: Cold Spring Harbor Laboratory Press Label: Cold Spring Harbor Laboratory Press Publisher: Cold Spring Harbor Lab.

[17] Alexandre Melnikov, Peter Rogov, Li Wang, Andreas Gnirke, and Tarjei S. Mikkelsen. Comprehensive mutational scanning of a kinase in vivo reveals substrate-dependent fitness landscapes. *Nucleic Acids Research*, 42(14):e112–e112, August 2014.

[18] Parul Mishra, Julia M. Flynn, Tyler N. Starr, and Daniel N. A. Bolon. Systematic Mutant Analyses Elucidate General and Client-Specific Aspects of Hsp90 Function. *Cell Rep*, 15(3):588–598, April 2016.

[19] Fabian Pedregosa, Gael Varoquaux, Alexandre Gramfort, Vincent Michel, Bertrand Thirion, Olivier Grisel, Mathieu Blondel, Peter Prettenhofer, Ron Weiss, Vincent Dubourg, Jake Vanderplas, Alexandre Passos, and David Cournapeau. Scikit-learn: Machine Learning in Python. p. 6, 2011.

[20] Andrew F. Philip, Masato Kumauchi, and Wouter D. Hoff. Robustness and evolvability in the functional anatomy of a PER-ARNT-SIM (PAS) domain. *PNAS*, 107(42):17986–17991, October 2010. Publisher: National Academy of Sciences Section: Biological Sciences.

[21] Hangfei Qi, C. Anders Olson, Nicholas C. Wu, Ruian Ke, Claude Loverdo, Virginia Chu, Shawna Truong, Roland Remenyi, Zugen Chen, Yushen Du, Sheng-Yao Su, Laith Q. Al-Mawsawi, Ting-Ting Wu, Shu-Hua Chen, Chung-Yen Lin, Weidong Zhong, James O. Lloyd-Smith, and Ren Sun. A Quantitative High-Resolution Genetic Profile Rapidly Identifies Sequence Determinants of Hepatitis C Viral Fitness and Drug Sensitivity. *PLOS Pathogens*, 10(4):e1004064, April 2014. Publisher: Public Library of Science.

[22] Liat Rockah-Shmuel, Ágnes Tóth-Petróczy, and Dan S. Tawfik. Systematic Mapping of Protein Mutational Space by Prolonged Drift Reveals the Deleterious Effects of Seemingly Neutral Mutations. *PLOS Computational Biology*, 11(8):e1004421, August 2015. Publisher: Public Library of Science.

[23] Philip A. Romero, Tuan M. Tran, and Adam R. Abate. Dissecting enzyme function with microfluidic-based deep mutational scanning. *PNAS*, 112(23):7159–7164, June 2015. Publisher: National Academy of Sciences Section: Biological Sciences.

[24] Benjamin P. Roscoe and Daniel N. A. Bolon. Systematic Exploration of Ubiquitin Sequence, E1 Activation Efficiency, and Experimental Fitness in Yeast. *Journal of Molecular Biology*, 426(15):2854–2870, July 2014.

[25] Benjamin P. Roscoe, Kelly M. Thayer, Konstantin B. Zeldovich, David Fushman, and Daniel N. A. Bolon. Analyses of the Effects of All Ubiquitin Point Mutants on Yeast Growth Rate. *Journal of Molecular Biology*, 425(8):1363–1377, April 2013.

[26] Lea M. Starita, Jonathan N. Pruneda, Russell S. Lo, Douglas M. Fowler, Helen J. Kim, Joseph B. Hiatt, Jay Shendure, Peter S. Brzovic, Stanley Fields, and Rachel E. Klevit. Activity-enhancing mutations in an E3 ubiquitin ligase identified by high-throughput mutagenesis. *PNAS*, 110(14):E1263–E1272, April 2013. Publisher: National Academy of Sciences Section: PNAS Plus.

[27] Lea M. Starita, David L. Young, Muhtadi Islam, Jacob O. Kitzman, Justin Gullingsrud, Ronald J. Hause, Douglas M. Fowler, Jeffrey D. Parvin, Jay Shendure, and Stanley Fields. Massively Parallel Functional Analysis of BRCA1 RING Domain Variants. *Genetics*, 200(2):413–422, June 2015. Publisher: Genetics Section: Communications.

[28] Michael A. Stiffler, Doeke R. Hekstra, and Rama Ranganathan. Evolvability as a function of purifying selection in TEM-1 -lactamase. *Cell*, 160(5):882–892, February 2015.

[29] Nicholas C. Wu, C. Anders Olson, Yushen Du, Shuai Le, Kevin Tran, Roland Remenyi, Danyang Gong, Laith Q. Al-Mawsawi, Hangfei Qi, Ting-Ting Wu, and Ren Sun. Functional Constraint Profiling of a Viral Protein Reveals Discordance of Evolutionary Conservation and Functionality. *PLOS Genetics*, 11(7):e1005310, July 2015. Publisher: Public Library of Science.

images/hmm.png

Figure 1: Hidden Markov Model with $z_k = (t_{k-1}, t_k)$ for Pattern Matching prediction

images/hmm2.png

Figure 2: Hidden Markov Model with $z_k = (C_k, t_{k-1}, t_k)$ for Pattern Matching Inference
